# Reviews

**Published:** 1862-05

**Authors:** 


### REVIEWS.

*The Principles and Practice of Obstetrics. By* Gunning S. Bedford, A. M., M. D., *Prof. of Obstetrics, the Diseases of Women and Children, and Clinical Obstetrics, in the University of New York; author of "Clinical Lectures on the Diseases of Women and Children," Illustrated by four Colored Lithographic Plates and ninety-nine Wood Engravings. Second edition, carefully revised.* 8vo. *pp.* 763. *New York:* Samuel S. & William Wood, 389 *Broadway,* 1862.

The appearance of the second edition of this work in the short interval of four months, is certainly a very remarkable fact, when we consider the usually rather slow sale of medical works of even great excellence. No better proof of its undoubted excellence could be desired by the author. Its very favorable reception has not been limited to this country, for foreign writers and reviewers, have not been slow to award to it, the highest praise. We do not doubt that it is entitled to be considered the best book on Obstetrics which has been published in the English language. A careful re-examination of this edition confirms the opinion of its merits, which we formerly expressed, viz: that it will be found a most complete, scientific, admirably arranged, and carefully considered book. One cannot, upon a thorough reading of it, fail to be impressed with its soundness and value, and we wish our readers to feel this as thoroughly as we do. Besides this we would direct attention to its attractions as a literary composition. A book which may contain much that is valuable, may be utterly unreadable, from bad arrangement, or faulty style, and the method may prove so repulsive as to make one entirely unwilling to get at the matter, however good. Dr. Bedford's work has this, we may say, if not remarkable, at least not very common recommendation, that it is written in an attractive style, and is therefore what a good book, and particularly a good text-book, should be, scientific, sound and *readable.*

We know of no work in which the whole subject of scientific and practical obstetrics has been so thoroughly treated. Upon examination it will be found that nothing within the range of our present knowledge, bearing upon the subject, has been omitted, or has failed to receive sufficient attention. The arrangement, moreover, is rigidly systematic, the various subjects being considered in the order which is most useful to the student. Although the book is not lacking in any particular, which properly pertains to a thorough consideration of the science of Obstetrics, it is a thoroughly practical work, suitable alike to the practitioner and the student. It contains ample evidence of a ripe and wise experience—one which has distrusted novelties, but has been progressive enough to gather up of the new whatever was of value. New methods of treatment, which have the sanction of great names, receive attention from the author, but they are not adopted by him, merely because they have the approval of eminent authority. The author is evidently very decided in his methods, though eminently candid.

We have no hesitation in declaring the work one of unsurpassed excellence; certainly no book with which we are acquainted, among the many excellent books lately published in this department, commends itself to our mind so completely in all respects.

---

*Anatomy, Descriptive and Surgical, by* HENRY GRAY, F. R. S., *Fellow of the Royal College of Surgeons, and Lecturer on Anatomy at St. George's Hospital Medical School. The drawings by* H. V. CARTER, M. D., *late Demonstrator of Anatomy at St. George's Hospital. The dissectings, jointly, by the author and* DR. CARTER. *Second American from the revised and enlarged London edition, with three hundred and ninety-five engravings on wood. Philadelphia:* BLANCHARD & LEA.

Gray's Anatomy needs no very extended notice from us. Every student of Anatomy *must*, as a matter of necessity, have this book. Every surgeon must have it, as a book altogether indispensable, since the drawings which represent the surgical regions are so accurate and reliable that too much cannot be said in its favor. It takes the place of the recent subject and keeps the relation of parts as freshly in the mind as can be done by any representations. To the student of anatomy it far surpasses all other books, since every artery, nerve, and vein are represented in position; the muscles are beautifully and truthfully represented, showing origin and insertion. And the bones are also shown in their different surfaces, with their lines, processes, articulations, &c. The value of all this is greatly increased by the names of the different parts being placed in situ upon the illustration, thus showing the part and at the same time giving the anatomical name. A brief microscopic anatomy of many of the tissues, and of the various organs, has also been introduced.

The appearance of the second American edition of Gray's extensive and comprehensive Anatomy is at this time particularly gratifying, and it will no doubt meet an extensive and ready sale. It is printed and bound in the usual style of Blanchard & Lea's publications, the beauty and stability of which are unsurpassed.

*A Book about Doctors, by* J. Cordy Jefferson. *Reprinted from the English edition. New York:* Rudd & Carlton, 130 *Grand Street. London:* Hurst & Blackett.

The book before us does not, as we might at first infer from its title, treat solely of those great physicians of the past, whose names all regular physicians have delighted to honor, but it touches as well upon famous quacks, both men and women, who at some portion of their lives attained a success almost unbounded. It not only gives us biographies of Sir Thomas Browne, Sir Hans Sloane, Drs. Radcliffe, Mead and Lettsom; but it contains accounts of noted quacks, such as Valentine Greatrakes, who cured by "shaking of hands;" St. John Long, whose marvellous lotion could distinguish between sound and unsound tissues, and Mrs. Mapp, the natural bonesetter. Some of the chapters are more general in their subjects, for instance, those on "Early English Physicians," "Imagination as a remedial power," and the "Country Medical Man.'

The author has exhibited great industry in collecting from a broad field' anecdotes and details touching the lives of celebrated physicians, and has succeeded in giving us a very pleasant and attractive book. It is certainly very pleasant to be able to learn how famous doctors lived, what good things they said, how they eat, drank, loved fees and practical generosity, hid a kind heart under a rough manner, quarrelled, kept good company, and fell in love like other mortals. Upon the whole we may feel that they had with all their faults a fine humanity, being cheerful, generous and humane, as to the world at large, reserving all their animosity and venom, if any they had, for each other. The quarrels of doctors have generally led to a vigorous use of the pen, rather than the pistol; though when duels have taken place, happily but rarely, they have been of the most sanguinary character. As regards the doctor as a man, our respect is rather increased by the pleasant narrative of our author. We notice that the notion of physicians being inclined to infidelity—a most unfounded one—is as old as Chaucer, who says of his physician, that "his study was but lytyl in the Bible." There is this consolation to be drawn from the accounts of regular physicians and quacks, that the former enjoyed their fame and success to the end of their lives, whereas the prosperity of quacks has been mainly short lived.

We think this will be found to be a very pleaeant and instructive book, and will serve to occupy agreeably the few leisure hours which fall to the lot of a hard-worked physician. We feel grateful to the author for enabling us to give to those to whom they belong certain anecdotes and witticisms which have heretofore had a rather uncertain paternity.

---

*Amputation of the Cervix Uteri, by* J. MARION SIMS, M. D., *Surgeon to the Womans' Hospital, New York. (Extracted from the Transactions of the Medical Society of the State of New York,* 1861.*)*

In this pamphlet a new mode of operation is advocated and illustrated by wood cuts. "The operation is performed by "splitting the cervix laterally, and removing first the anterior, and then the posterior half, by means of scissors. After the hæmorrhage has ceased, the vaginal mucus membrane is drawn over the stump and secured by four silver sutures, two on each side of the cervical opening. In the old method of amputation the suppuration continued five or six weeks; sometimes longer, before the parts were entirely cicatrized. According to this plan there is no suppuration, the parts healing by the first intention, and the patient becoming well in a week. In the former, there was danger of pyæmia; in this, there is little, if any. In the former there was risk of degeneration of tissue; in this, there is none; in the former, the parts contracted as they healed; in this, they remain normal. It might have been supposed, *a priori,* that this operation would have been attended by a troublesome hæmorrhage, but in each case it ceased as soon as the vaginal tissue was drawn over the stump, and in no instance was a ligature used."

Six cases operated upon since the publication of the Transactions of the State Medical Society are also reported in detail. The paper is written in good style; is modest and unostentatious, and gives a favorable impression poth of the operation and of the author's inventive genius and operative skill.

---

*London Lancet. A journal of British and Foreign Medicine, Physiology, Surgery, Chemistry, Literature, Criticism and News.* JAMES HERLD, *American Publisher,* 14 *Ann St., New York.*

This is the most valuable monthly reprint in this country, and deserves the support of the profession, while the profession also very much need its support; it is a benefit which "works both ways." It presents a great variety of subjects, and all papers admitted to its pages are carefully studied and thoroughly prepared; nothing but the choicest material is allowed place. Those in the active practice of medicine and surgery will find it eminently practical in character; while at the same time the science and theory of diseased action are fully considered. It commends itself to the profession in an eminent degree, and probably receives a wider circulation than any other medical journal.

